# Testing a Spanish-language colorectal cancer screening decision aid in Latinos with limited English proficiency: Results from a pre-post trial and four month follow-up survey

**DOI:** 10.1186/1472-6947-12-53

**Published:** 2012-06-12

**Authors:** Daniel S Reuland, Linda K Ko, Alicia Fernandez, Laura C Braswell, Michael Pignone

**Affiliations:** 1 Division of General Medicine and Clinical Epidemiology, University of North Carolina, Campus Box 7110, Chapel Hill, NC 27599, USA; 2 Cecil G. Sheps Center for Health Services Research, University of North Carolina, Campus Box 7590, Chapel Hill, NC 27599, USA; 3 Lineberger Comprehensive Cancer Center, University of North Carolina, Campus Box 7295, Chapel Hill, NC 27599, USA; 4 Fred Hutchinson Cancer Research Center, University of Washington, 1100 Fairview Ave. North, M3-B232, Seattle, WA 98109, USA; 5 Division of General Internal Medicine, University of California, SFGH Bldg. 10, Ward 13, San Francisco, CA 94143, USA

**Keywords:** Decision aid, Latinos, Limited English proficiency, Colorectal cancer screening

## Abstract

**Background:**

Compared with non-Latinos, Latinos in the US have low rates of colorectal cancer (CRC) screening and low rates of knowledge regarding CRC screening tests and guidelines. Spanish speaking Latinos have particularly low CRC screening rates and screening knowledge. Our purpose was twofold: (1) to evaluate the effect of a computer-based, Spanish-language CRC screening decision aid on screening knowledge, intent to obtain screening, and screening self-efficacy in a community sample of Latinos with limited English proficiency (LEP); and (2) to survey these decision aid viewers at four months to determine their rates of CRC discussions with a health care provider as well as their rates of screening test completion.

**Methods:**

We recruited 50-75 year old Latinos with LEP who were not current with CRC. Participants screening viewed a 14 minute multimedia decision aid that addresses CRC screening rationale, recommendations, and options. We conducted an uncontrolled (pre-post) study in which we assessed screening knowledge, self-efficacy, and intent at baseline and immediately after decision aid viewing. We also conducted a follow-up telephone survey of participants at four months to examine rates of patient-provider screening discussions and test completion.

**Results:**

Among n = 80 participants, knowledge scores increased from 20% (before) to 72% (after) decision aid viewing (absolute difference [95%CI]: 52% [46, 59]). The proportion with high screening self-efficacy increased from 67% to 92% (25% [13, 37]); the proportion with high screening intent increased from 63% to 95% (32% [21, 44]). We reached 68 (85%) of 80 participants eligible for the follow-up survey. Of these 36 (53%) reported discussing screening with a provider and 13 (19%) completed a test.

**Conclusion:**

Viewing a Spanish-language decision aid increased CRC screening knowledge, self-efficacy, and intent among Latinos with LEP. Decision aid viewing appeared to promote both CRC screening discussions with health care providers and test completion. The decision aid may be an effective tool for promoting CRC screening and reducing screening disparities in this population.

## Background

Colorectal cancer (CRC) is the second leading cause of cancer mortality in the US. National guidelines recommend that individuals over age 50 receive colorectal cancer screening [1,2]. Although screening rates have increased in recent years, important screening disparities exist for ethnic and racial minorities. CRC screening rates in Hispanic/Latino populations, now the nation’s largest and fastest growing racial/ethnic minority group, are among the lowest nationally [3,4]. Latinos are also more likely to be diagnosed with advanced stage CRC than non-Hispanic whites and have a lower probability of survival after diagnosis [5,6]. Latinos who have limited English proficiency (LEP) represent a vulnerable population. Compared with Latinos who are proficient in English, LEP Latinos tend to have lower levels of formal education and literacy, experience challenges to communication in health care settings, and lack awareness of CRC screening guidelines [7-14]. Latinos with LEP have lower CRC screening rates than Latinos who are proficient in English, [9,10] and having LEP is an independent risk factor for lack of screening even after accounting for multiple socio-economic and healthcare access factors [8,11,15].

Multimedia patient education tools have the potential to improve communication about CRC screening for vulnerable populations, including those with LEP. Multimedia formats may be particularly helpful in overcoming literacy barriers by having text read aloud by a narrator and through the use of graphics and animations [16]. Because they can be delivered outside of the patient-provider encounter, multimedia educational tools may also help to overcome provider-level communication barriers such as lack of time to educate patients about screening. Decision aids in particular may promote screening adherence by more explicitly incorporating patients’ preferences into colorectal cancer screening decisions. Studies in English speaking populations have found that CRC screening decision aids can increase patients’ knowledge and intent to obtain screening and may increase screening test completion [16-20]. However, few studies have evaluated multimedia CRC screening educational interventions in Spanish speaking Latinos, a population with low health literacy and often low formal education, and no studies have evaluated an actual CRC screening decision aid in Spanish speakers [21-23]. Further, no studies have assessed whether individuals from this population who view a decision aid outside of a clinic setting subsequently discuss CRC screening with a health care provider or complete a recommended CRC screening test.

In this article we report the findings of two study phases. The objective of the first phase was to determine the effect of viewing a Spanish-language CRC screening decision aid on CRC screening-related knowledge, self-efficacy, and intent among screening-eligible Latinos with LEP. The objective of the second phase was to assess the rates of CRC screening discussions with health providers and screening test completion at four months after decision aid viewing.

## Methods

### Participant recruitment and eligibility

We recruited a convenience sample from both community and clinic registry sources. Community recruitment was passive and included Spanish-language fliers posted on community bulletin boards and regional Spanish-language newspaper advertisements. Clinic registry recruitment was conducted through queries of patient registration data at two sites: a Federally Qualified Health Center (FQHC) in Caswell County, NC, and an academic medical center in central NC. The queries identified Hispanic/Latino individuals aged 50-75 years old who were then recruited using a mailing and follow-up telephone call. Recruiting materials, including fliers and mailings, described the research as being related to “health education materials” and to “cancer prevention”. However, the materials did not include terms specific to CRC such as “colon cancer”. After the mailing, we recruited some participants via a follow-up phone call inquiring about their interest in participating*.* To be eligible for the study, participants had to be 50-75 years old, report their ethnicity as “Hispanic” or “Latino”, report that they speak English less than “very well”, have a preference to receive health care information in Spanish, have average risk for CRC (i.e. have no family history of CRC or personal history of precancerous polyps), and not be current with CRC screening defined as having had either a colonoscopy within the last 10 years or a fecal occult blood test (FOBT) within the past year.

#### Intervention description

##### Decision aid development and initial message testing

We developed the Spanish-language decision aid using a formative research process aimed at producing a cultural and linguistic adaptation of a previously developed and tested English language decision aid [17,19]. Both the Spanish and English language decision aids are based on existing behavior theories including Prochaska’s Transtheoretical Model [24] and Social Cognitive Theory [25]. As part of the adaptation process, we convened focus groups from the target community to identify relevant socio-cultural perspectives and themes regarding CRC screening and CRC communication with their doctors. Emerging themes included embarrassment about screening procedures, views of physicians as authority figures, familism (a tendency to place a high value on the central position that the family holds in the life of the individual), personalism (a tendency to value the person-to-person connection highly), machismo, and language barriers. We also used focus groups to engage members of the target population directly in the process of incorporating these themes into the Spanish-language version of the decision aid. In pre-testing the decision aid for content and usability, we found high levels of trust, comprehension, agreement, and relevance among 18 individuals who met the above-noted eligibility criteria. A more detailed description of this formative research process and a qualitative analysis of focus group data is the subject of a separate manuscript [26].

##### Decision aid content and format

**Figure 1:**
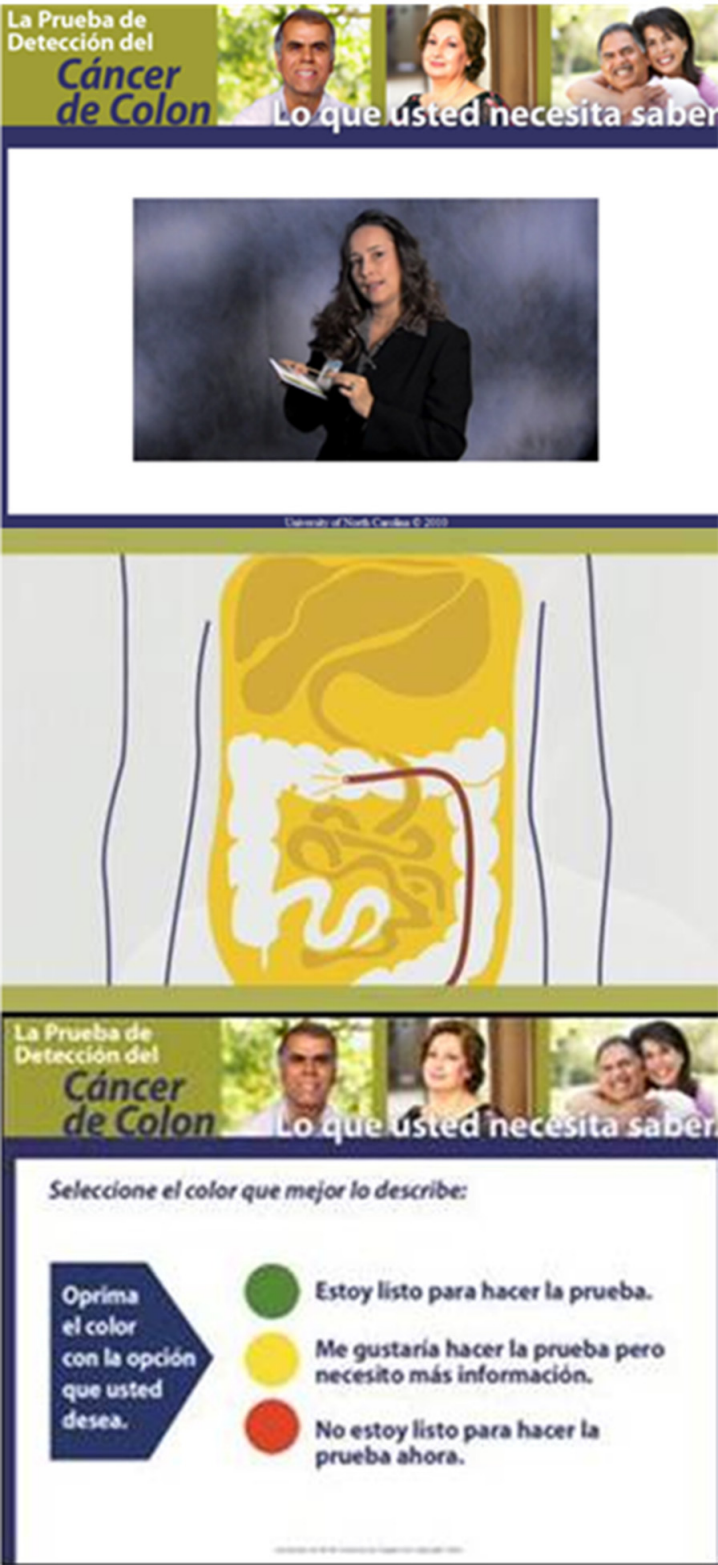
Decision aid screen shots.

The decision aid was a multimedia intervention that included a 14-minute Spanish-language video (see Figure 1 for sample screen shots) plus a printed brochure. The video could be viewed via web-streaming or on a DVD player. The content included an overview and rationale for CRC screening, specific information about colonoscopy and FOBT (currently the most widely available, guideline-recommended screening tests), vignettes from patients about their decision to be screened and why they chose a particular screening test, and a summary of key characteristics of the two screening tests, including test frequency, cost, overall effectiveness, time required, discomfort, and risk of complications. A table comparing these key characteristics of the screening modalities is reviewed orally by the narrator in the video.

The decision aid was designed to be accessible regardless of literacy level. All written text was read aloud by a narrator, and technical terms and concepts were explained using easy-to-understand narration, vignettes, graphics, and animations. At the end of the video, viewers were prompted to select one of three pre-printed, color brochures corresponding to their readiness for screening. The brochures used a “traffic light” color coding scheme with the green brochure indicating readiness to be screened (preparation for action stage), the yellow brochure indicating considering becoming screened (contemplation stage), and the red brochure indicating that the viewer was not considering screening (pre-contemplation stage) (Figure 1). The brochures also included the above noted table comparing the screening options. After a brochure was selected, viewers were promoted to show the brochure to their physician and to discuss their preferences and readiness for screening. A copy of the decision aid, including the brochures, can be obtained from the authors by visiting the decision aid website [27].

##### Data collection

Participants were enrolled by telephone and completed a baseline questionnaire during the initial phone contact. The baseline questionnaire assessed CRC screening knowledge, intent to be screened, and self-efficacy, demographic characteristics, and preferences for shared decision making [28]. After completing the baseline questionnaire, participants were scheduled to view the decision aid in person. Decision aid viewing was not linked to clinical visits with health care providers. Participants viewed the decision aid individually in a private conference room located at either the clinical site from which they were recruited, our research facility, or a local public library in the presence of a research assistant. After viewing the decision aid, participants completed a second oral (face-to-face) survey that measured screening knowledge, self-efficacy, and intent. We also surveyed participants by telephone four months after viewing the decision aid to determine whether they had discussed CRC screening with a health care provider and/or completed a screening test.

All questionnaires were administered orally in Spanish by bilingual research assistants. Data were collected from October 2010 through January 2012. The study was approved by the institutional review board at the University of North Carolina at Chapel Hill. Participants received a $50 gift card following the completion of the in-person decision aid viewing appointment and survey, and a $20 gift card for completion of telephone surveys.

#### Measures

##### Phase 1 outcome measures

The Phase 1 (pre-post) outcomes were screening-related knowledge, self-efficacy, and intent assessed immediately after viewing the decision aid. *Knowledge* was assessed by a six item index that assessed the following content areas: 1) the availability of more than one option for CRC screening; 2) the availability of a home screening test; 3) the recommended age to begin CRC screening; 4) FOBT screening test frequency; 5) the need for sedation (and companion driver) for colonoscopy procedure, and 6) the existence of a small but non-zero complication risk associated with having colonoscopy. The knowledge items, which were developed by the investigators based on decision aid content, were in a true-false format with a third response option of “don’t know” also offered.

Intent and self-efficacy measures were adapted from measures used in prior CRC screening studies [17,20,29]. *Intent* to become screened in the next six months was assessed using a single categorical item with three response options (definitely planning to be screened, considering being screened, not considering being screened). *Self-efficacy* was assessed using a single categorical item asking participants how sure they were that they could become screened in the next six months (sure, a little unsure, very unsure). Spanish language versions of the intent and self-efficacy outcome measures were pre-tested in n = 18 members of the target population in a previous study phase, along the knowledge items described above.

### Phase 2 outcome measures

Outcomes for the four month follow-up telephone survey were participant-reported rates of 1) having had a CRC screening discussion with a health care provider, 2) having received a recommendation for specific CRC screening test(s), and 3) whether a CRC screening test was completed. These items were adapted from items used in a nationally representative survey that included Spanish speakers [30].

#### Statistical analysis

We calculated descriptive statistics (means and percentages) from the baseline survey to characterize the study population. We then conducted further analysis aimed at determining the efficacy of the decision aid in changing participants’ screening related knowledge, self-efficacy and intent before versus after decision aid viewing. For CRC screening knowledge, we dichotomized the six individual knowledge item responses as either correct or incorrect by treating “don’t know” responses the same as incorrect responses. We treated the total knowledge score (% of items answered correctly) as a continuous variable and calculated the mean change (absolute difference) in this score from before to after viewing the decision aid. We also calculated the 95% confidence interval (CI) for this difference. We tested whether mean pre and post knowledge scores differed statistically using a paired *t*-test.

We dichotomized the categorical screening *intent* variable into high (“definitely planning to be screened”) versus lower (other responses). Similarly, we dichotomized the screening *self-efficacy* variable as higher (“sure”) versus lower (“a little/very unsure”). For these categorical variables, we calculated the difference in proportions before versus after, as well as 95% confidence intervals for the difference, and we tested whether the pre and post proportions differed statistically using a McNemar test.

For the follow-up survey outcomes, we calculated the proportions of respondents who reported they had CRC screening discussions, received specific test recommendations, and completed screening tests. Analyses were conducted using Stata version 11.2 (College Station, TX).

## Results

We enrolled 80 participants whose characteristics are summarized in Table 1. Fifty seven percent were recruited via the community ads, and the remaining 43% were recruited from the clinical registry sources. The mean age of participants was 56 years, and 64% were female. Two-thirds were from Mexico and Central American countries, and most (91%) spoke English either “not very well” or “not at all”. A majority (61%) had household incomes under $20,000; two-thirds were uninsured, and three-fourths had less than high school education. A majority of participants (58%) either did not use or were “uncomfortable” using computers. Most indicated a preference for either an active (49%) or shared role (35%) in medical decisions.

**Table 1:** Participant Characteristics (N = 80) Mean (SD) or %

Age in years	56 (± 4.9)
Country of Origin	
Mexico	45
Central America	21
South America	29
Caribbean	5
Sex (Female)	64
Speaks English	
Very well	0
Well	10
Not very well	58
None	33
Years in the US	
<11	38
11-20	39
>20	24
Insurance	
Uninsured	66
Private	23
Public (Medicare or Medicaid)	8
Other/unsure	4
Education	
<8 years	41
8-12 years	35
13+ years	24
Employed full-time	29
Recruitment Source	
Community	57
Clinical Registry	43
Household income < $20,000	61
Overall Health	
Excellent/very good/good	44
Fair/poor	56
Comfort using a computer	
Very/somewhat comfortable	42
Very/somewhat uncomfortable	17
Don’t know how to use	41
Awareness of Colorectal Cancer	
Heard of Colon Cancer	88
Heard of Polyp	35
Heard of FOBT	43
Decision Control Preferences	
Active	49
Shared	35
Passive	16
Doctor ever recommended FOBT	
Yes	18
No	82

*FOBT* = fecal occult blood testing.

### Phase I outcomes: Screening knowledge, self-efficacy, and intent

**Figure 2:**
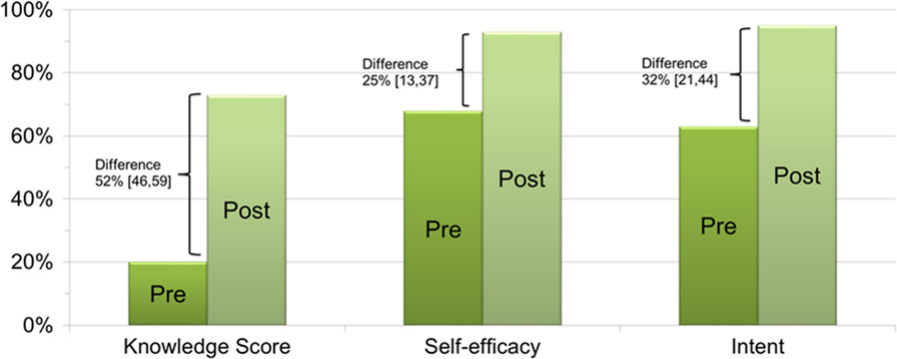
**Pre-post measures of knowledge, self-efficacy and intent (n = 80)**. Legend for Figure 2: Knowledge score equals percent correct on six items. Self-efficacy represents the proportion of participants reporting that they were “sure” they could be screened in the next six months versus “a little”/“very unsure”. Screening intent equals the proportion of participants who reported they were “definitely planning” to be screened within the next six months versus those who were “thinking about” or “not considering” screening.

Pre-post changes in screening related knowledge, self-efficacy, and intent are shown in Figure 2 (n = 80). Baseline CRC screening knowledge scores were low and increased from 20% to 72% after viewing the decision aid; (absolute difference = 52% [95%CI 46, 59]). Viewing the decision aid was associated with increases in all knowledge content areas (Table 2). For the outcome of CRC screening self-efficacy, the proportion of participants reporting they were “sure” they could be screened increased from 67% to 92% (difference 25% [13,37]). With respect to CRC screening intent, the proportion of participants reporting high intent (i.e. “definitely planning”) to become screened from 63% to 95% (difference 32% [21, 44)]. All differences were statistically significant at p < 0.001.

**Table 2:** Knowledge regarding CRC screening before vs. after viewing the decision aid (N = 80)

**Outcome**	**% Correct**		Change*	**95% CI**
	**Pre**	**Post**		
Knowledge Score	20%	72%	+52%	[46,59]
There is only one test for CRC screening (*false*)	14%	60%	+46%	[34,59]
It is not possible to do a CRC screening test at home (*false)*	24%	84%	+40%	[48,72]
Age to begin screening (50 years old)	44%	84%	+40%	[27,54]
FOBT is done every 3 years (*false)*	6%	65%	+59%	[46,71]
One can drive to work immediately after colonoscopy (*false*)	28%	75%	+47%	[35,61]
No risks to colonoscopy procedure (*false*)	6%	69%	+63%	[51,74]

*p < 0.0001 by paired *t* test for knowledge score.

p < 0.0001 McNemar test for individual items.

#### Screening test preferences

After decision aid viewing 42 (52%) of viewers preferred FOBT, 35 (44%) preferred colonoscopy, and 4% said they were unsure.

### Phase 2, follow-up survey results

We reached 68 (85%) of 80 participants for the four month follow-up survey. Of these respondents 57 (84%) had seen a health care provider since viewing the decision aid, 36 (53%) reported discussing screening with a provider, 24 (35%) recalled receiving a specific recommendation or referral to have a screening test from the provider, and 13 (19%) said they had completed a screening test. Of these 13, seven completed FOBT, five completed colonoscopy, and one reported completing both tests. Because only 13 completed a screening test, analysis of correlates of screening test completion was not performed due to small cell sizes.

## Discussion

We found that viewing a Spanish-language decision aid for CRC was associated with increased screening-related knowledge, self-efficacy, and intent among Latinos with LEP, demonstrating that the information provided by the decision is accessible and compelling to this target population. These findings suggest that such a decision aid may be useful as an effective means of communicating this relatively complex message content about evidence-based cancer screening guidelines to a vulnerable population outside of an actual health care provider encounter.

In our telephone follow up survey, we also found that more than half of respondents had discussed CRC screening with a health care provider within four months of decision aid viewing. While our study lacked a comparison group against which to directly compare these figures, data from nationally representative surveys suggests that such discussions occur infrequently. In one survey, less than one third of Spanish speaking Latinos had discussed CRC screening with a physician within the past two years [31]. Interventions such as this that can promote informed discussions between patients and health care providers about screening represent one promising means of addressing screening disparities since patient provider communication plays a key role in promoting CRC screening. Other studies have found that receiving a physician recommendation for CRC screening is an independent predictor of adherence to CRC screening guidelines in the general population, [7,8,32] and suboptimal communication between patients and providers is likely one of several factors contributing to screening disparities in Latinos [9].

In our follow-up survey, we also found that one in five respondents actually completed a screening test, with more than half completing a FOBT. Although this study lacked a direct comparison group, other evidence suggests it is unlikely that 19% of unscreened members of this target population would become current with CRC screening in any four month period in the absence of an intervention [4,11,15]. Being current with CRC screening requires having had a colonoscopy anytime in the past 10 years (or FOBT within one year), and only about 33% of Spanish speaking Latinos are current with CRC screening [15].

To our knowledge, this is the first study to describe patient-reported clinical communication and screening behavior after viewing a CRC screening decision aid in this population. This is also the first study to assess the extent to which individuals from this population who view such a CRC screening tool outside of a clinic setting subsequently discuss CRC screening with a health care provider or complete a recommended CRC screening test. Thus, our study complements and extends findings from a limited number of other studies of multimedia CRC screening educational interventions in Spanish speaking Latinos. One other study found that an educational video about CRC improved patient knowledge about CRC in Spanish speaking patients; however, that study did not ascertain subsequent clinical communication or screening behavior [21]. Another promising study conducted in 2009 in an urban teaching hospital found that screening among Spanish speaking Latinos was more likely after receiving an intervention that included an educational video; however the study was small (31 patients received the intervention) and the researchers did not collect data from patients regarding their communication with physicians [22].

This decision aid intervention addresses barriers to suboptimal patient-provider communication and low screening rates in limited English proficiency (LEP) Latino populations. These barriers include literacy issues, brief primary care patient-provider visits in which competing demands from other health issues result in lack of time to fully counsel patients about screening, inadequate numbers of Spanish speaking health care providers, and poor integration of trained interpreters into primary care service delivery [7,13,33-35]. Nevertheless, the fact that only a minority of viewers actually completed a screening test suggests that important questions still remain about how to achieve high levels of adherence to screening guidelines and the role that multimedia patient education tools such as decisions aids have in promoting screening in these populations. Other studies of CRC screening decision aid interventions in English speaking patient populations have also found that while decision aids help patients become informed and activated regarding screening, screening rates remain low because other barriers interfere with actual completion of screening tests [16,20,29]. These other barriers can include lack of access to insurance or financial assistance to cover the cost of colonoscopy (either as the primary screening test or to follow up an abnormal FOBT test), transportation challenges, lack of reliable telephone access, unpredictable work schedules, child care responsibilities, lack of understanding how to complete a home stool test, and difficulties with colonoscopy preparation procedures [10,12-14,34]. Similarly, our findings suggest that although this decision aid can help patients become informed and activated regarding CRC screening and can promote clinical discussions, it is likely that additional interventions that address other barriers to CRC screening test completion are needed to effectively promote screening and eliminate screening disparities in vulnerable populations. Individualized one-on-one support such as patient navigation to help patients overcome other practical barriers as well as policies or programs that address access barriers to colonoscopy will likely be needed in order to eliminate disparities in CRC screening [36,37].

Our study has some limitations. Because we used a one group (pre-post) design without a separate control group, we are unable to determine whether ongoing efforts by local or regional organizations to promote CRC screening influenced screening behavior among our participants. We are also unable to determine how a less intensive intervention, such as written information about CRC screening, would have affected outcomes in this population. However, the provision of written materials alone has had very little if any effect as a single intervention in studies conducted in English speaking populations, [37] and given low educational and literacy levels in our target population, it is likely that providing written material alone would have little impact on communication or screening. Second, our follow-up survey outcomes relied on participant self-report and may be subject to recall bias. Third, our study used a convenience sample recruited from a single region and our results may not be generalizable to other LEP Latino communities regionally or nationally. Nevertheless, our observed effects were large and our sample included diverse Latino communities and countries of origin. Hence, we believe the decision aid should be tested in larger studies involving Latino populations and communities.

## Conclusions

In conclusion, this study suggests that this decision aid is efficacious in educating Latinos who have LEP about CRC and activating them to communicate with health care providers about screening. Overall, this decision aid could function as an effective component of a CRC screening intervention aimed at addressing disparities in CRC screening for LEP Latinos.

## Abbreviations

CRC: Colorectal cancer; LEP: Limited English proficiency; FOBT: Fecal occult blood test; FQHC: Federally qualified health center.

## Competing interests

The authors declare that they have no competing interests.

## Authors’ contributions

DR was the principle investigator. DR, AF, MP were involved in the initial conception and design of the study. DR, LK and LB implemented and carried out the actual study, including the instrument development, refinement, and translation, as well as data collection. DR performed statistical analysis with input from all authors. DR and LB drafted the manuscript with substantial review and input from all authors. All authors read and approved the final manuscript.

## Pre-publication history

The pre-publication history for this paper can be accessed here:

http://www.biomedcentral.com/1472-6947/12/53/prepub
